# Rethinking glutamine metabolism and the regulation of glutamine addiction by oncogenes in cancer

**DOI:** 10.3389/fonc.2023.1143798

**Published:** 2023-03-07

**Authors:** Rui Ni, Ziwei Li, Li Li, Dan Peng, Yue Ming, Lin Li, Yao Liu

**Affiliations:** ^1^ Department of pharmacy, Daping Hospital, Army Medical University, Chongqing, China; ^2^ Department of pharmacy, Women and Children’s Hospital of Chongqing Medical University, Chongqing Health Center for Women and Children, Chongqing, China

**Keywords:** glutamine metabolism, oncogene, glutamine addiction, metabolism inhibiton, cancer therapy

## Abstract

Glutamine, the most abundant non-essential amino acid in human blood, is crucial for cancer cell growth and cancer progression. Glutamine mainly functions as a carbon and nitrogen source for biosynthesis, energy metabolism, and redox homeostasis maintenance in cancer cells. Dysregulated glutamine metabolism is a notable metabolic characteristic of cancer cells. Some carcinogen-driven cancers exhibit a marked dependence on glutamine, also known as glutamine addiction, which has rendered the glutamine metabolic pathway a breakpoint in cancer therapeutics. However, some cancer cells can adapt to the glutamine unavailability by reprogramming metabolism, thus limiting the success of this therapeutic approach. Given the complexity of metabolic networks and the limited impact of inhibiting glutamine metabolism alone, the combination of glutamine metabolism inhibition and other therapeutic methods may outperform corresponding monotherapies in the treatment of cancers. This review summarizes the uptake, transport, and metabolic characteristics of glutamine, as well as the regulation of glutamine dependence by some important oncogenes in various cancers to emphasize the therapeutic potential of targeting glutamine metabolism. Furthermore, we discuss a glutamine metabolic pathway, the glutaminase II pathway, that has been substantially overlooked. Finally, we discuss the applicability of polytherapeutic strategies targeting glutamine metabolism to provide a new perspective on cancer therapeutics.

## 1 Introduction

Metabolic reprogramming is a hallmark of cancer that manifests in several ways. Cancer cells exhibit substantially enhanced glucose uptake and full utilization of the glycolysis/tricarboxylic acid (TCA) cycle to generate a large amount of ATP (Warburg effect) (1). Additionally, cancer cells may exhibit accelerated uptake, transport and metabolism of glutamine, which is the most abundant non-essential amino acid in the human body. Adaptation to rapid metabolism is achieved by regulating genes that encode metabolic drivers to increase the expression of favorable transporters and metabolic enzymes. Furthermore, metabolism-regulating interactions occur between cancer cells and the tumor microenvironment (TME) (2). Since Hans Krebs first studied glutamine metabolism in animals, the biological roles of glutamine in cellular growth and cancer cell biology have gradually been recognized (3). In 1955, Eagle discovered a high dependence of cancer cells on glutamine, also known as glutamine addiction (4). Specifically, cancer cells consume 10–100-fold more glutamine than they do any other amino acids. Glutamine is the most abundant non-essential amino acid in the bloodstream. High levels of glutamine can promote cancer cell proliferation by serving as nitrogen and carbon sources for the biosynthesis of nucleotides, fatty acids, and non-essential amino acids. In addition to its primary roles in macromolecular biosynthesis, glutamine is also involved in the cellular uptake of essential amino acids, the maintenance of the mitochondrial membrane potential, and the production of glutathione and nicotinamide adenine dinucleotide phosphate (NADPH), which are required to maintain redox homeostasis (5–7). Altered glutamine metabolism is a significant outcome of changes in energy metabolism in cancer cells. Abnormal expression of regulatory genes associated with glutamine metabolism is more frequently observed in cancer cells than in healthy cells. Most abnormally expressed regulatory genes, including B-Raf proto-oncogene (*BRAF*), epidermal growth factor receptor (*EGFR*), isocitrate dehydrogenase 1 (*IDH1*), Kirsten rat sarcoma viral oncogene homolog (*KRAS*), and phosphatidylinositol 3-kinase p110 alpha (*PIK3CA*), are either oncogenes or tumor suppressor genes involved in the onset and development of cancer (8). Mutations in some of these oncogenes render cancer cells highly dependent on glutamine. Given the critical biologica roles of glutamine in cancer cells, an in-depth understanding of glutamine metabolism is essential to develop new cancer treatments.

Therefore, this review summarizes the uptake, transport, and metabolic characteristics of glutamine, as well as the regulatory effects of some important oncogenes on glutamine addiction in cancer to emphasize the therapeutic potential of targeting glutamine metabolism. In particular, we discuss the significance of the glutaminase II pathway, which is a historically understudied glutamine metabolic pathway in the context of cancer. Furthermore, we also discuss the potential applications of polytherapy that targets glutamine metabolism to provide novel strategies for treating cancer.

## 2 Glutamine metabolism in cancer

**Figure 1:**
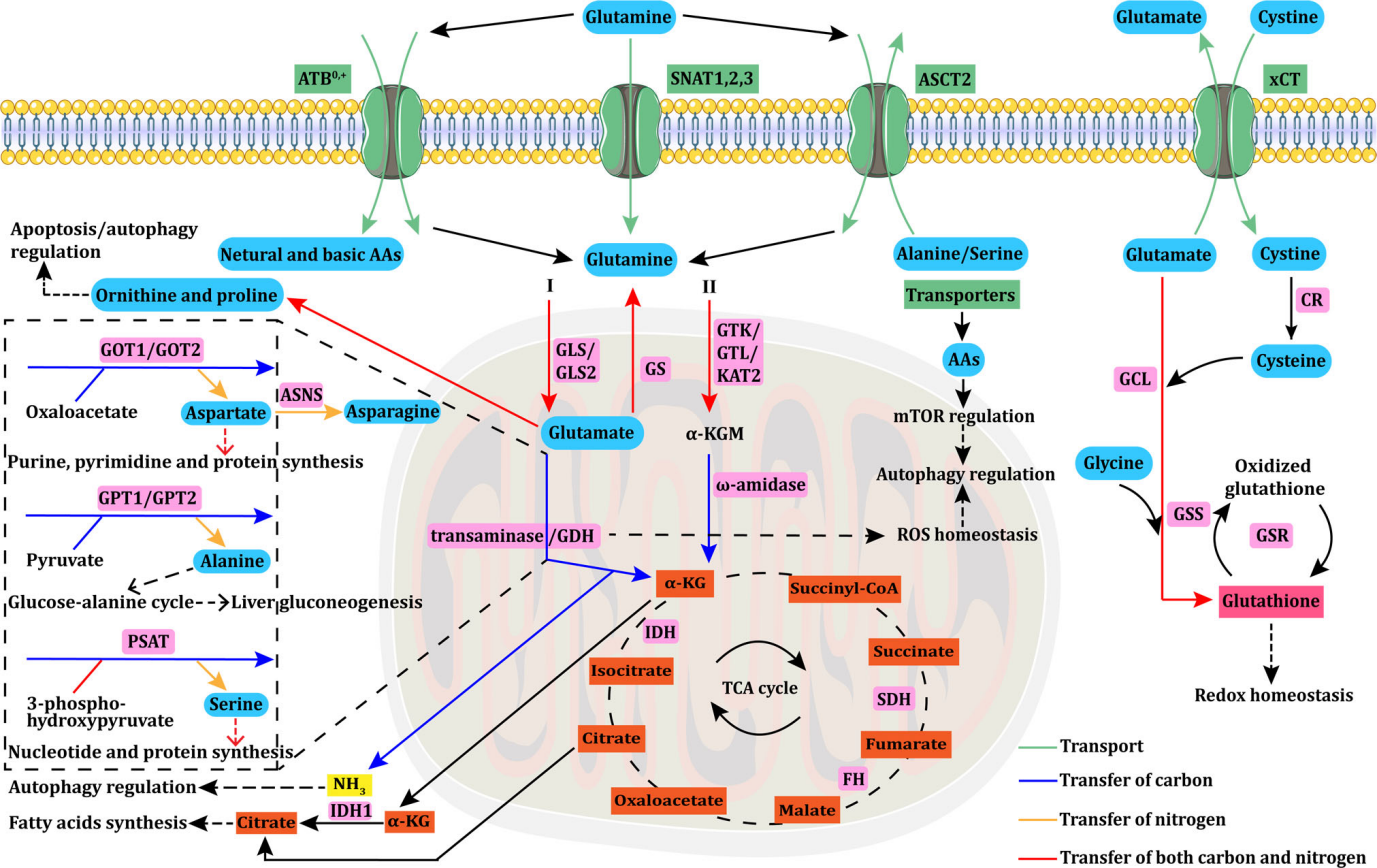
Glutamine uptake, transport, and metabolism. Glutamine uptake and transport in cancer cells is mediated by transporters (ASCT2/ATB^0,+^/SNAT1,2,3). Intracellular glutamine can be converted into α-KG through the glutaminase I (GLS+GDH/transaminase) or II (GTK/GTL/KAT2+ω-amidase) pathway. Aspartate, alanine, and serine are mainly produced *via* transaminases, such as GOT, GPT, and PSAT. GOT transforms glutamate into aspartate, which is essential for purine, pyrimidine, and protein synthesis. Aspartate is further converted to asparagine *via* ASNS. GPT catalyzes the reversible conversion of glutamate to pyruvate for α-KG and alanine generation. This GPT-catalyzed reaction plays a vital role in the glucose–alanine cycle, which is essential to support liver gluconeogenesis. PSAT transforms glutamate into serine, which is involved in nucleotide and protein synthesis. α-KG is an important anaplerotic substrate of the TCA cycle, in which FH, IDH, and SDH mutations are the main causes of cycle dysfunction and mitochondrial metabolic defects in various types of cancers. Glutamine can be synthesized from glutamate *via* GS. Glutamine can also be converted to ornithine and proline, which regulate apoptosis/autophagy. The byproduct of glutaminolysis, ammonia, can modulate autophagy under specific circumstances. The reductive carboxylation of α-KG into citrate can support adipogenesis, which is crucial for cancer cell survival. The GDH pathway is also linked to cellular ROS homeostasis, and further modulates autophagy, which can also be regulated by AAs through the mTOR pathway. Glutamate is exchanged for cystine *via* xCT, which is rapidly reduced to cysteine by CR. Then, glutamate, cysteine, and glycine react together for *de novo* synthesis of glutathione *via* GCL and GSS. The interconversion of oxidized glutathione and glutathione is catalyzed by GSR, and glutathione can directly control redox homeostasis. AAs, amino acids; ASNS, asparagine synthetase; α-KG, alpha-ketoglutarate; CR, cystine reductase; GCL, glutamylcysteine ligase; GSS, glutathione synthetase; GSR, glutathione reductase; GLS, glutaminase; GDH, glutamate dehydrogenase; GS, glutamine synthase; GTK/GTL, glutamine transaminases K/L; KAT2, kynurenine aminotransferase 2; GOT, glutamate-oxaloacetate transaminase; GPT, glutamate-pyruvate transaminase; PSAT, phosphoserine aminotransferase; FH, fumarate hydratase; IDH, isocitrate dehydrogenase; SDH, succinate dehydrogenase; ROS, reactive oxygen species.

Metabolism, a fundamental process for all cellular functions, is also related to cancer cell proliferation. Unlike normal differentiated cells, cancer cells can modify many metabolic pathways—including glycolysis, glutaminolysis, the TCA cycle, the electron transport chain, and the pentose phosphate pathway—to fulfill their energy requirements (2). Following the discovery of the Warburg effect, numerous studies have confirmed the crucial role of cancer cell metabolism in tumor survival and growth. However, recent studies have demonstrated that glutamine plays a more significant role in cancer metabolism than was previously thought. The glutamine demand is usually higher in cancer cells than in normal cells owing to accelerated glutamine metabolism. Despite being a non-essential amino acid and the most abundant amino acid in humans, glutamine is considered to be conditionally essential in some instances due to its involvement in multiple cellular processes (9). Below, we summarized the uptake, transport, and functions of glutamine, and its relevant metabolic enzymes (**Figure 1**).

### 2.1 Glutamine uptake and transport in cancer cells

Glutamine uptake by cancer cells is mediated by transporters (10). Alanine-Serine-Cysteine Transporter 2 (ASCT2), a sodium (Na^+^)-dependent transmembrane transporter encoded by the solute carrier family 1 member 5 (*SLC1A5*) gene, mediates the cellular uptake of glutamine and other neutral amino acids and is considered to be a primary glutamine transporter in cancer cells (11, 12). L-type amino acid transporter 1 (LAT1), which is encoded by the *SLC7A5* gene, preferentially transports large branched and aromatic neutral amino acids, and its expression is upregulated in various types of cancer cells (13, 14). Intracellular glutamine can serve as an exchange substrate to import extracellular amino acids, including leucine, isoleucine, valine, phenylalanine and tyrosine *via* LAT1 with high affinity (15). In addition, intracellular glutamine can be metabolized into glutamate, which serves as an exchange substrate for cystine uptake mediated by the cystine/glutamate transporter xCT (*SLC7A11*). The imported cystine can then be converted to cysteine for the biosynthesis of glutathione and the maintenance of cellular redox homeostasis (16). ASCT2, LAT1, and xCT are antiporters that balance cytosolic amino acid composition by proportionally exchanging intracellular amino acids with extracellular amino acids. Hence, these antiporters do not mediate affect intracellular glutamine levels unless other amino acids are available for exchange.

Since the net movement of amino acids into cells can only be mediated by uniporters and symporters (17–19), their expression may also be crucial for cellular growth. Neutral amino acid transporters belong to the SLC38 gene family of sodium-coupled neutral amino acid transporters (SNATs), among which SNAT1 (*SLC38A1*) and SNAT2 (*SLC38A2*) are uniporters (19). Both SNAT1 and SNAT2 play important roles in the net uptake of glutamine in epithelial cervical cancer cells, osteosarcoma cells, human melanoma cells, and six breast cancer cell lines (19–21). The first member of the SLC38 family to be cloned was SNAT3 (*SLC38A3*) (22). Unlike SNAT1 and SNAT2, SNAT3 preferentially imports glutamine, asparagine, and histidine (23). SNAT3 is overexpressed in malignant glioma and non-small cell lung cancer (NSCLC) (24, 25). The amino acid transporter B^0,+^ (ATB^0,+^), a symporter encoded by the *SLC6A14* gene, can transport all neutral (including glutamine) and basic amino acids. ATB^0,+^ is overexpressed in various types of cancer, including colorectal (CRC), breast, and pancreatic cancers (26). Additional information on glutamine transporters is provided in **Table 1**.

**Table 1:** Main membrane-anchored amino acid transporters of glutamine, their properties, and the cancers in which they are overexpressed.

Solute carrier number	Common name	Na^+^-dependence (Transporter type)	Substrates	Overexpression in cancers
SLC1A5	ASCT2	Yes (antiporter)	Ala, Ser, Cys Gln, Thr, Asn	breast (27), colorectal (28), Eesophageal (29), gastric (30), lung (31), ovarian (32), and prostate (33) cancers; neuroblastoma (34); endometrial (35), renal cell (36), and hepatocellular (37) carcinomas; head and neck (38), oral (39), and esophageal (40) squamous cell carcinomas
SLC7A5	LAT1	No (antiporter)	large branched-chain and aromatic neutral AAs	breast (41), biliary tract (42), colorectal (43), gastric (44), lung (45), pancreatic (46), and prostate (47) cancers; leukemia (48); glioblastoma (49); melanoma (50); ovarian (51) and hepatocellular (52) carcinomas; esophageal (40), oral (53), and laryngeal (54) squamous cell Carcinomas
SLC7A11	xCT	No (antiporter)	Glu, Cystine	breast (55), colorectal (56), and ovarian (57) cancers; melanoma, adrenocortical, bladder, renal cell (58), and hepatocellular (59) carcinomas; lung adenocarcinoma (60); glioblastoma multiforme (61); head and neck squamous cell carcinoma (62)
SLC38A1	SNAT1	Yes (uniporter)	small, neutral AAs	breast (63), colorectal (64), gastric (65), lung (66), and ovarian (67) cancers; melanoma (20); leukemia (68); hepatocellular carcinoma (69)
SLC38A2	SNAT2	Yes (uniporter)	Ala, Asn, Cys, Gln, Gly, His, Met, Pro, Ser	breast (21), colorectal (70), gastric (71), lung (66), and prostate (72) cancers
SLC38A3	SNAT3	Yes (uniporter)	Gln, Asn, His	malignant gliomas (24); non-small cell lung cancer (25)
SLC6A14	ATB^0,+^	Yes (symporter)	neutral and basic AAs	colorectal (73), gastric (74), pancreatic (75), and estrogen receptor-positive breast (76) cancers; cervical carcinoma (77)

AAs, amino acids; Ala, alanine; Asn, asparagine; Cys, cysteine; Gln, glutamine; Glu, glutamate; Gly, glycine; His, histidine; Met, methionine; Pro, proline, Ser, serine; Tyr, tyrosine.

### 2.2 Glutamine metabolism in cancer cells

#### 2.2.1 Epigenetics and the dysregulation of the tricarboxylic acid cycle

Here, we address the dysregulated TCA cycle in cancer cells before summarizing glutamine metabolism in cancer cells. The TCA cycle is an intracellular central metabolic hub and a common catabolic pathway for various types of sugar, amino acids, and fatty acids (78). TCA cycle dysregulation can directly alter the metabolism of cancer cells. Alpha-ketoglutarate (α-KG), which is derived from glutamine catabolism, is an important anaplerotic substrate of the TCA cycle.

Mutations in genes involved in the TCA cycle are associated with familial cancers (79). Mutations in the genes encoding fumarate hydratase (FH), isocitrate dehydrogenase (IDH), and succinate dehydrogenase (SDH) cause TCA cycle dysfunction and mitochondrial metabolic defects in various types of cancers (80). Mutations occur throughout the FH gene, and heterozygous mutations in this gene are related to dominantly inherited uterine fibroids, hereditary leiomyomatosis, and renal cell carcinoma (81, 82). FH acts as a tumor suppressor, and downregulation of its expression results in the accumulation of hypoxia-inducible factor-1α (HIF-1α) and fumarate (83). Fumarate is an oncometabolite and a potent inhibitor of prolyl 4-hydroxylase (P4H), which is a negative regulator of HIF-1α. Inhibition of P4H leads to HIF-1α activation under normoxic conditions, resulting in pseudohypoxia that promotes the proliferation of cancer cells (79). IDH has three isoforms: IDH1, IDH2, and IDH3. Genomic analyses have revealed IDH1 or IDH2 mutations in samples from most patients with glioblastoma multiforme and grade II-III gliomas (84). Furthermore, mutations in IDH1 at arginine 132 (R132) and in IDH2 at arginine 172 (R172) have also been identified in patients with acute myeloid leukemia (85). Virtually almost all IDH1 and IDH2 mutations are missense mutations located at R132 and R172, respectively; both isotypes are therefore promising biomarkers for cancer diagnosis and gene therapy. Mutations in IDH cause the loss of its enzymatic activity of converting isocitrate to α-KG while conferring a neo-enzymatic activity of reducing α-KG to the oncometabolite D-2-hydroxyglutarate, an excess of which favors the formation of malignant tumors (86). Similar to FH mutations, heterozygous mutations in the SDH gene are associated with hereditary paraganglioma and pheochromocytoma (87). In addition, mutations in genes encoding SDH subunits are found in other types of tumors, such as gastrointestinal stromal tumor (88), testicular seminoma (89), neuroblastoma (90), renal cell carcinoma (91), and thyroid cancer (92).

#### 2.2.2 Functions of glutamine

##### 2.2.2.1 Glutamine as a nitrogen donor

Glutamine is first converted by the glutaminase I (GLS/GLS2) system to glutamate, which is then converted to α-KG through two pathways. One pathway is mediated by the enzymatic activity of glutamate dehydrogenases (GDH). The other pathway is mediated by glutamate-oxaloacetate transaminase (GOT), glutamate-pyruvate transaminase (GPT), and phosphoserine aminotransferase (PSAT), which catalyze the conversion of glutamine to α-KG without producing ammonia. Additionally, glutamine can also be converted into α-KG through the glutaminase II pathway, which is described in detail below. Transaminases catalyze the biosynthesis of amino acids such as aspartate, alanine, and serine using the amino group of glutamine. GOT, also known as aspartate transaminase, has cytoplasmic (GOT1) and mitochondrial (GOT) isoforms. GOT catalyzes the conversion of glutamate to aspartate, which is required for biosynthesizing purines, pyrimidines, and proteins, as well as maintaining redox homeostasis by the malate–aspartate shuttle (93). Prominent studies demonstrate that aspartate biosynthesis is an essential function of mitochondrial respiration in proliferating cells (94, 95). In view of the multiple critical roles of aspartate, aspartate uptake and biosynthesis pathways have been are considered therapeutic targets in cancer (93). GPT, also called alanine transaminase, includes the cytoplasmic GPT1 and mitochondrial GPT2 isoforms and catalyzes the reversible conversion of glutamate to pyruvate for α-KG and alanine generation. This GPT-catalyzed reaction plays an important role in the glucose–alanine cycle, which is essential for supporting hepatic gluconeogenesis (96). PSAT is required to produce serine, which plays a key role in the biosynthesis of nucleotides and proteins and is an allosteric activator of several enzymes. Approximately half of all α-KG that enters the TCA cycle in breast cancer cell lines is produced *via* the activity of PSAT (97); thus, serine biosynthesis may play an important role in cancer cell metabolism. Glutamine can also be converted to ornithine and proline. Proline dehydrogenase/proline oxidase-dependent apoptosis/autophagy may be modulated by the interconversion of glutamate, ornithine, and proline, among which proline is the key amino acid (98). Understanding the regulatory roles of the proline in this process could facilitate the development of targeted cancer therapies. Glutamine also serves as a nitrogen donor for the biosynthesis of asparagine catalyzed by asparagine synthase (ASNS). In addition to amino acid biosynthesis, glutamine also provides nitrogen for the *de novo* synthesis of purines, pyrimidines, and nucleobases of DNA and RNA (99).

##### 2.2.2.2 Glutamine as a carbon donor

One of the most important metabolic pathways involving glutamine is the biosynthesis of α-KG to provide an anaplerotic supply for the TCA cycle. α-KG can be metabolized *via* oxidative decarboxylation and reductive carboxylation. Hypoxia (100), impaired mitochondrial respiration (101), and anchorage-independent formation of tumor spheroids (102) can promote the reductive carboxylation of glutamine-derived α-KG into citrate to support adipogenesis, which is crucial to cancer cell survival. Under such circumstances, glutamine serves as a direct carbon source for citrate and fatty acid biosynthesis (103, 104).

##### 2.2.2.3 Reactive oxygen species homeostasis and autophagy regulation

Glutamine produces reactive oxygen species (ROS) in addition to providing nitrogen and carbon for biosynthesis and energy metabolism. However, some glutamine metabolites can directly control ROS levels. For instance, glutathione can neutralize peroxide free radicals, and NADPH generated *via* GDH-catalyzed glutamine metabolism can regulate ROS homeostasis by directly scavenging excess ROS (105). Glutamine also plays an important regulatory role in autophagy suppression through various processes affected by its metabolism. ROS promote autophagy in response to stress. Meanwhile, glutamine modulates autophagy through the production of glutathione and NADPH, which affect ROS levels (106). Additionally, glutamine is involved in the activation of mammalian target of rapamycin (mTOR), which inhibits autophagy. The latest research demonstrates that glutaminolysis is one of two mechanisms through which glutamine metabolism modulates the mTORC1-autophagy pathway (107). A byproduct of glutaminolysis, ammonia, induces and inhibits autophagy at low and high concentrations, respectively (108). Thus, ammonia regulates autophagy in a concentration-dependent manner without relying on inhibited mTOR or ULK1/2 activity (108, 109). The GDH-catalyzed conversion of glutamate to α-KG generates a second molecule of ammonia in some types of cancer cells. However, others preferentially use transaminases for this reaction, thereby bypassing the generation of the second ammonia molecule. Changes in this metabolic pathway could thus indirectly affect autophagy regulation.

### 2.3 Enzymes involved in glutaminolysis

#### 2.3.1 Glutaminase

Greenstein discovered the two glutaminase enzyme systems in rats (110). The glutaminase I system, which is now known as the glutaminase or phosphate-activated glutaminase system, comprises kidney (GLS or KGA) and liver (GLS2 or LGA) isozymes activated by phosphate (111), while the glutaminase II system is activated by α-keto acids (112).

The mitochondrial glutaminase I system converts imported glutamine to glutamate and is considered the first key enzymatic reaction in the catalysis of glutamine in cancer cells. The ubiquitous expression of GLS in normal tissues is upregulated in various cancers, and it might play a key role in cancer onset and progression (113). GLS has thus been extensively explored as a target for drug development. In contrast, GLS2 is primarily expressed in the liver, brain, pituitary gland, and pancreas. Whether GLS2 promotes or suppresses tumorigenesis remains uncertain, and it has rarely been targeted in drug discovery (111, 114). However, GLS significantly promotes cancer cell growth. The expression of GLS is abnormally elevated in multiple cancer types, including breast (115), colorectal (116), and prostate (117) cancers, especially when compared with adjacent tissues. Knocking down or inhibiting GLS can restrict the growth of various types of cancer cells and suppress cancer progression (113, 115, 118). The GLS inhibitor CB-839 is currently entering phase I and II clinical trials as monotherapy and as polytherapy when combined with chemotherapy and/or immunotherapy.

Glutaminase II is an enzymatic complex comprising glutamine transaminase and omega (ω)-amidase (119, 120). The main glutamine transaminases in humans and rodents are glutamine transaminases K (GTK) and L (GTL) (121, 122). Kynurenine aminotransferase 2 (KAT2), also exerts some enzymatic action on glutamine (123). In the presence of various α-keto acids, glutamine transaminases catalyze the conversion of glutamine into alpha-ketoglutaramate (α-KGM), which is then deamidated by ω-amidase into α-KG. To date, cancer cell glutamine addiction is still widely and naturally thought to involve the glutaminase I pathway, by which glutamine is first converted to glutamate *via* a GLS-catalyzed reaction and before being converted into α-KG through GDH or transaminases (GOT/GPT/PSAT). However, the glutaminase II pathway can also realize the conversion of glutamine into α-KG. This pathway’s functions in cancer cells have been substantially overlooked, with only a few relevant studies having been reported. For instance, it has been newly confirmed that the glutaminase II pathway exists in human pancreatic cancers, and genetic suppression of GTK completely inhibits pancreatic tumorigenesis *in vivo* (124). Almost concurrently, the glutaminase II pathway was identified in prostate cancer cells, and expression of the GTK and ω-amidase genes in this pathway has been shown to be more upregulated with increased cancer cell invasiveness (125). The glutaminase II pathway also plays important roles in providing anaplerotic carbon to the TCA cycle, supplying citrate carbon in prostate cancers, and closing the methionine salvage pathway (125). These findings suggest that glutamine transaminase (GTK) and ω-amidase could be novel metabolic targets for cancer treatment. However, the broad substrate specificity of glutamine transaminase towards α-keto acids and amino acids should be considered. Inhibiting glutamine transaminase might interfere with other biological properties. Additionally, glutamine metabolism through the glutaminase I pathway is notably catalyzed by GLS+GDH requires an aerobic environment due to the involvement of nicotinamide adenine dinucleotide (NAD^+^). In contrast, α-KG production through the glutaminase II pathway does not involve net oxidation, indicating that it can function in hypoxic regions of tumors. In summary, the glutaminase II pathway in cancers has provided a new perspective for studying cancer cell glutamine addiction and for developing corresponding therapeutic strategies.

#### 2.3.2 Glutamate dehydrogenase

Glutamate dehydrogenase (GDH) catalyzes the second step in glutamine metabolism, which is the oxidative deamination of glutamate into α-KG. The GDH1 isozyme is expressed ubiquitously across tissues and cells, whereas GDH2 is specifically expressed in brain, testis, and embryonic tissues (126). Aside from producing anaplerotic α-KG for the TCA cycle, the GDH pathway is also associated with various cellular processes, including acid–base balance, ammonia metabolism, lactate production, redox homeostasis, and lipid biosynthesis (127). Specifically, recent research indicates that GDH is crucial for metabolic ammonia recycling in breast cancer cells, especially estrogen-receptor-positive cells, to support their growth and proliferation (128). Additionally, GDH1 plays a vital role in maintaining redox homeostasis in breast and lung cancer (129). GDH1 expression is significantly upregulated in tumor samples from patients with advanced breast or lung cancer, resulting in the accumulation of fumarate. Fumarate can bind to and activate the ROS-scavenging enzyme glutathione peroxidase 1 (GPx1), conferring metabolic advantages for cancer cell growth and proliferation. The small-molecule inhibitor R162, which targets GDH1, can reduce the proliferative capacity of cancer cells by causing redox homeostasis imbalance (129). The survival of glioblastoma cells with glucose metabolism disorders due to glucose deprivation, glycolytic inhibition, or Ak strain transforming (Akt) signaling inhibition requires GDH (130). Overexpressed GDH promotes the proliferation, migration, and invasion of CRC cells, and might serve as a novel independent prognostic biomarker for CRC progression and metastasis (131). Glutamine enhances the proliferative capacity of ovarian cancer cells in a dose-dependent manner and increases the activities of GLS and GDH by modulating the mTOR/ribosomal S6 kinase (S6) and mitogen-activated protein kinase 1 (MAPK) pathways (132). GDH expression is upregulated in extrahepatic cholangiocarcinoma tissues, whereas silencing it significantly reduces the proliferative, migratory, and invasive capacity of cancer cells. Hence, GDH is considered an important prognostic marker and therapeutic target in extrahepatic cholangiocarcinoma (133).

The roles of GDH2 in cancer growth and metabolism have not been fully investigated. However, GDH2 plays an important role in eliminating the growth-inhibiting effect of IDH1 (R132H) mutant gliomas (134). This suggests that targeting GDH2 could be a beneficial strategy for treating patients with IDH1 mutant gliomas.

#### 2.3.3 Transaminase

GOT1/GOT2, GPT1/GPT2, and PSAT are important enzymes in glutamine metabolism and amino acid biosynthesis, and have crucial functions in various types of cancers (135). GOT1-mediated pathways play vital roles in maintaining redox homeostasis in pancreatic cancer, and increased enzymatic activity of GOT1 favors the growth of cancer cells (136). A recent study demonstrates that inhibiting GOT1 activity hinders the growth of several pancreatic ductal adenocarcinoma cell lines, primary tumor models, and tumor xenografts (137). GOT2 acetylation is essential for regulating the mitochondrial NADH/NAD^+^ ratio and stimulating the production of NADPH to maintain the redox state of pancreatic cancer cells (138). Acetylation of GOT2 at the K404 lysine residue promotes the proliferation of pancreatic cancer cells and tumor growth *in vivo*, and GOT2 acetylation at the lysine residue K159 is increased in human pancreatic tumors. Triple-negative breast cancer (TNBC) cell lines also overexpress GOT1, which controls intracellular ROS levels by producing NADPH, in turn promoting tumor growth. The shRNA-mediated inhibition of GOT1 expression enhances cancer cell sensitivity to doxorubicin by causing doxorubicin-induced ROS generation (139). The transaminase inhibitor aminooxyacetate (AOA) can inhibit the proliferation of breast cancer cells (140).

The enzymatic activity of GPT2 is pivotal to the anchorage-independent growth of KRAS-transformed colon cancer cells (141). PIK3CA mutations can trigger glutamine metabolism reprogramming in CRC cells by upregulating GPT2 expression (142). GPT2 overexpression enhances the tumorigenicity and stemness of breast cancer cells by activating the sonic hedgehog signaling pathway, suggesting its potential as a therapeutic target (143).

Recent studies show that elevated ratios of aspartate to alanine transaminases (AST/ALT or GOT/GPT) correlate with a poor prognosis in bladder (144), colorectal (145), hepatic (146), head and neck (147), oral and oropharyngeal (148), prostate (149), and pancreatic (150) cancers. The metabolic roles of PSAT in cancer cells have not yet been comprehensively explored. It has been reported that significantly more PSAT is expressed in colon tumors than in normal tissues (151), but its specific mechanism and prognostic value remain to be elucidated.

#### 2.3.4 Glutamine synthetase

Glutamine can be synthesized *de novo* from glutamate *via* glutamine synthase (GS). The selective, irreversible GS inhibitor L-methionine sulfoximine (MSO) has anticancer potential due to its ability to inhibit cancer cell proliferation *in vitro* (152, 153).

## 3 Regulation of glutamine addiction by oncogenes in cancer

Cancer-associated genes are broadly categorized as oncogenes and tumorsuppressor genes that promote and suppress carcinogenesis, respectively. Interactions between these two types of genes result in oncogenesis (154). Hence, cancers are ultimately outcomes of dysregulated gene expression. Genes can be activated or inactivated by mutations. Some of the prominent mutated oncogenes in cancers include *BRAF*, *ErbB2*, *JAK2*, *KRAS*, *MYC*, and *PIK3CA*. Mutations in these oncogenes can result in a high reliance of cancer cells on glutamine for survival and proliferation. Therefore, depleting glutamine and inhibiting glutamine metabolism eventually lead to the growth arrest or even death of these glutamine-addicted cancer cells. Below we summarize the regulatory effects of some important oncogenes on glutamine addiction in cancers.

### 3.1 *BRAF* mutation

The *BRAF* gene is one of three rapidly accelerated fibrosarcoma (RAF) isoforms that encodes the serine/threonine kinase of the RAS family. The *BRAF* mutation rate in all cancers is about 7%, but the rate varies, being about 66% in melanoma and 10%–25% in CRC (155). Over 90% of mutations in the *BRAF* gene occur in codon 600; the substitution of valine (V) with glutamic acid (E) (BRAF V600E) is the most common mutation, followed by the substitution of V with lysine (K) (BRAF V600K) (156). Therefore, the National Comprehensive Cancer Network (NCCN) recommends that patients with advanced CRC should be tested for *BRAF* mutations before starting first-line treatment. Targeted polytherapies for patients with mutated BRAF include the triplet regimens of Dabrafenib (BRAF inhibitor) + Trametinib (MEK inhibitor) + Panitumumab or Cetuximab and Encorafenib (BRAF inhibitor) + Binimetinib (MEK inhibitor) + Panitumumab or Cetuximab.

Melanoma cells, which contain mutated BRAF and are resistant to the BRAF inhibitor PLX4720, exhibit increased oxidative metabolism and mitochondrial dependence for survival (157). The increased oxidative metabolism is related to a shift from glucose to glutamine metabolism and a greater dependence on glutamine over glucose. Such cells are more sensitive to glutamine metabolism inhibitors and mitochondrial poisons. Hence, combining inhibitors of BRAF and glutamine metabolism has a more prominent therapeutic effect. Melanoma cell lines with single resistance to Vemurafenib (BRAF inhibitor) and double resistance to Vemurafenib/Selumetinib (MEK inhibitor)exhibit increased glutamine uptake and NH4+ production without changes in glucose uptake (158). Furthermore, glutamine deprivation induces the apoptosis of drug-resistant cell lines. Taking advantage of glutamine addiction, the glutaminase inhibitor BPTES and the glutamine-mimetic antimetabolite L-DON have yielded superior antitumor effects *in vivo.*

### 3.2 *KRAS* mutation

*KRAS*, *HRAS* and *NRAS*, which are three isoforms of the mammalian rat sarcoma (RAS) GTPase, encode KRAS4A, KRAS4B, HRAS, and NRAS. These four small G proteins bind to GTP/GDP and exhibit GTP hydrolase activity. As a molecular switch, RAS activates downstream signaling pathways, such as the MAPK and PI3K-Akt pathways, by binding to GTP to regulate the proliferation, differentiation, and apoptosis of cells. RAS mutations account for one-third of all human cancers (159, 160). The analysis of data retrieved from four cancer databases (COSMIC, cBioPortal, ICGC, and TCGA) reveals significantly higher mutation rates for *KRAS* than for *HRAS* and *NRAS* (160). A *KRAS* mutation is an important driver leading to metabolic reprogramming in cancer cells, and it is closely related to glutamine and its metabolic changes.

*KRAS* mutations increase glutamine demand in cancer cells that use glutamine to accelerate energy metabolism and maintain redox homeostasis (161). The selectively upregulated expression of ASCT2, LAT1 and SNAT2 in CRC cells containing mutated *KRAS* enhances the uptake of glutamine, leucine, and other amino acids. Knocking out *KRAS* downregulates the expression of the above amino acid transporters, thereby reducing the uptake of amino acids by CRC cells (70). Metabolomic analyses have revealed higher concentrations of amino acids, especially glutamine, in CRC cells containing mutated *KRAS* than in wild-type CRC cells (162). A subsequent study of amino acid transporters in CRC cells with mutated KRAS found that KRAS signaling mainly regulated the expression of ASCT2 through the PI3-Akt-mTOR pathway. Additionally, the prognosis of patients with mutated KRAS and high ASCT2 expression is worse than patients with mutated KRAS and low ASCT2 expression (28). A monoclonal antibody antagonist, Ab3-8, has been developed that recognizes the extracellular domain of ASCT2, reduces cellular glutamine import and Akt/ERK phosphorylation in SW1116 and HCT116 human CRC cell lines containing mutated KRAS, and inhibits the growth of tumor xenografts in mice (163). However, Ab3-8 does not inhibit the *in vivo* growth of tumor xenografts of the HT29 human CRC cells with wild-type KRAS. These findings collectively suggest that ASCT2 could be a useful target for treating cancers with mutated KRAS.

The effects of oncogenic KRAS mutations on glutamine metabolism have been confirmed. In human breast cancer cells with mutated KRAS, anabolic glutamine utilization is increased, and the expression of genes associated with glutamine metabolism is significantly upregulated, and there exists a high dependence on glutamine for cellular growth (164). Oncogenic KRAS promotes glutamine metabolism reprogramming, and glutamine deprivation can increase ROS levels and decrease reductive glutathione levels in pancreatic cancer cells (136). The anaplerotic feeding of the glutaminolysis metabolite α-KG into the TCA cycle is essential for the growth of various anchorage-independent, KRAS-induced cancer cells (141). In addition, LKB1 and KEAP1/NRF2 pathways synergistically promote metabolic reprogramming toward enhanced glutamine dependence in lung adenocarcinoma with mutated KRAS, and they also enhance the sensitivity of cancer cells to CB-839 (GLS inhibitor) *in vitro* and *in vivo* (165). Combining CB-839 with Selumetinib (MEK inhibitor) takes advantage of the increased glutamine utilization in NSCLC cells with mutated KRAS to improve therapeutic efficacy (166).

### 3.3 MYC activation

The myelocytomatosis (MYC) proto-oncogenes include *c-MYC*, *n-MYC*, *l-MYC*, and *r-MYC*, among which *c-MYC* is the most commonly activated proto-oncogene. As a transcription factor, MYC protein strictly responds to and integrates mitogenic and developmental signals into broad changes in gene expression to support cell growth and proliferation (167). In fact, most cancers harbor altered *MYC* genes. For instance, MYC is amplified in up to 78% of osteosarcomas, 65% of ovarian serous cystadenocarcinomas, 48% of breast cancers, 45% of esophageal cancers and 37% of lung squamous cell carcinomas (167). TCGA data show that MYC amplification accounts for 21% of all tumor samples (167). Furthermore, MYC signaling in cancer cells enables abnormal TME regulation and evasion of the host immune response. The inactivation of MYC in preclinical models might lead to sustained tumor regression due to oncogene addiction (168). Hence, MYC activation elicits numerous hallmarks required for autonomous tumor growth. Yuneva et al. first discovered that MYC-driven proliferating cells exhibit glutamine addiction (169). The expression of genes associated with glutamine metabolism can be positively stimulated by MYC. Sensitivity to glutamine deprivation is c-MYC-dependent in glioma cells and can be suppressed by targeting *MYC* expression (170). Furthermore, various cell lines derived from cancers such as P493-6 B-cell lymphoma, PC3 prostate cancer (171), osteosarcoma (172), Ramos and Raji B-cell lymphoma (173), renal cell carcinoma (174), HCT116 CRC (175), and U-1906 small cell lung carcinoma (176) cells rely on glutamine for cellular survival and growth under MYC activation. Interestingly, glutamine itself can also regulate c-MYC protein expression in HCT116 CRC (175), U266 and INA-6 multiple myeloma (177), and SK-N-AS and SH-SY5Y neuroblastoma (178) cells. Glutamine addiction extends from the context of MYC, and mutations in *KRAS* —a key gene related to the stability and activity of c-MYC protein— in cancer cells similarly cause dependence on exogenous glutamine for cellular growth and proliferation, as does MYC amplification (162, 179, 180). Therefore, combining therapy targeting the MYC pathway with intervention in glutamine metabolism should be key to reversing MYC-driven tumor growth and restoring the antitumor immune response.

### 3.4 mTORC1 activation

The mammalian target of rapamycin complex 1 (mTORC1) is a highly conserved protein kinase complex that regulates cellular growth, metabolism, and autophagy in response to exogenous signals from nutrients and growth factors (181). As a key downstream effector of many oncogenic pathways, mTORC1 is associated with cancer progression (182). Mutations in mTORC1 are often hyperactivating in cancer (183, 184), and mTOR inhibitors Rapalogs (rapamycin and its analogs) have been clinically approved by FDA for treating some cancers (185). Amino acids are likely required to activate mTORC1, which in turn can regulate amino acid metabolism (186, 187). With further research, activation of the mTORC1 pathway has been shown to be related to glutamine addiction in cancer cells (188). mTORC1 promotes glutamine anaplerosis by activating GDH in human epithelial cancer cell lines such as DLD1 colon cancer, as well as the prostate cancer cell lines LNCaP and DU145 (189). Mechanistically, mTORC1 promotes the proteasome-mediated destabilization of CREB2 to suppress SIRT4 (a mitochondria-localized member of the sirtuin family), which inhibits GDH, thereby enhancing GDH enzymatic activity. Furthermore, mTORC1 not only regulates GDH, but also promotes glutamine uptake by cancer cells by positively regulating GLS through S6K1-dependent c-MYC regulation (190). At the molecular level, S6K1 enhances the efficiency of MYC translation by regulating phosphorylation of the eukaryotic initiation factor eIF4B. The inhibitors of mTOR and GLS can significantly attenuate the growth of BxPC3 pancreatic cancer cells.

### 3.5 *PIK3CA* mutation

Phosphatidylinositol 3-kinase p110 alpha (*PIK3CA*) is an important proto-oncogene in the PI3K/Akt signaling pathway because it participates in the modulation of numerous cellular functions, including proliferation, differentiation, apoptosis, and glucose transport. It was initially detected by *in situ* hybridization, and is a 34-kb gene located in 3q26.3, which contains 20 exons (191). Among mutations in the *PIK3CA* gene, about80% occur in the helical and kinase domains; H1047R on exon 20 and E542K and E545K on exon 9 are the three most prevalent mutations (192). Mutations in *PIK3CA* play important roles in the onset and development of cancer and are found to have a rate of 2–7% in NSCLC, especially squamous cell lung carcinoma. *PIK3CA* mutations occur in CRC at a rate of 20–30% and often occur concomitantly with *KRAS* and *BRAF* mutations. Indeed, *PIK3CA* is one of the most commonly mutated genes, with a rate of about 30% in breast cancer (193). NCCN guidelines (2019) recommend *PIK3CA* mutation tests for patients with ER^+^/HER2^-^ breast cancer. Polytherapy with Alpelisib (PIK3CA inhibitor) and Fulvestrant (estrogen receptor antagonist) can improve the survival rates of patients with breast cancer contaning *PIK3CA* mutations.

Mutations in *PIK3CA* can reprogram glutamine metabolism by upregulating GPT2 expression, thereby increasing glutamine dependence of CRC cells. Specifically, the findings of metabolic flux analyses identified a higher rate of glutamine conversion to α-KG in CRC cells with mutated PIK3CA than in wild-type CRC cells. Mutations in the catalytic subunit p110α can upregulate GPT2 expression through the PDK1-RSK2-ATF4 signaling axis, thus increasing CRC cell dependence on glutamine. Blocking this signaling axis can inhibit the growth of CRC cells with mutated PIK3CA. The GPT2 inhibitor AOA can also inhibit the growth of CRC with mutated PIK3CA, but not in wild-type CRC xenografts (142). The results of [^13^C_5_]-glutamine tracer studies using mice with subcutaneous, orthotopic, and spontaneous CRC xenografts reveal that glutamine primarily enters the TCA cycle in tumors. Utilization rates of excess glutamine in tissue culture and subcutaneous xenografts, are higher for CRC with mutated PIK3CA than wild-type CRC. Levels of TCA cycle intermediates were shown to be more enriched in an orthotopic model with mutated PIK3CA, than in wild-type tumors (194). PIK3CA mutations induced in the MCF10A human mammary epithelial cell line result in a 50% increase in glutamine uptake and a significant increase in glutamate production (195). Therefore, *PIK3CA* mutations lead to glutamine addiction in tumors. Intervention in glutamine metabolism could facilitate treatment for cancers with mutated PIK3CA.

Given that CRC cells with mutated PIK3CA are more dependent on glutamine, CB-839 (GLS inhibitor) combined with 5-fluorouracil (5-FU) can significantly induce CRC cell apoptosis when compared with the corresponding monotherapies (196). The most recent results of an ongoing phase I clinical trial (NCT02861300) on CB-839 with Capecitabine (a 5-FU prodrug) for advanced CRC and other solid tumors yielded a better therapeutic outcome for patients with mutated PIK3CA than those with non-mutated cancers.

### 3.6 Other oncogenes

The activated proto-oncogene *ErbB2* (also termed *neu* or *HER2*) is a leading cause of breast cancer. More GLS mRNA and protein are expressed in *ErbB2*-transformed MCF10A than in MCF-10A cells, whereas *ErbB2* knockdown downregulates GLS expression. Further research has shown that activated ErbB2 stimulates GLS expression in breast cancer cells through the PI3K/Akt-independent NF-κB pathway (197). If these findings are validated in models *in vivo*, this could facilitate the identification of novel targets for cancer prevention and treatment.

A V617F mutation in the *JAK2* gene mutation is found in most patients with myeloproliferative neoplasms (MPNs). Among the peripheral blood cluster of differentiation CD34(+) cells from patients with MPN, more GLS is expressed in progenitor cells with mutated JAK2 than in progenitor cells with wild-type JAK2 [198]. More vigorous glutamine metabolism and significantly higher GLS expression are also confirmed in murine pro-lymphoid (BaF3) cells with mutated JAK2 when compared with that in cells with wild-type JAK2. Therefore, GLS inhibitors can improve the therapeutic effects of the JAK2 inhibitor Ruxolitinib by enhancing its inhibitory effect against CD34(+) and growth of cells with mutated JAK2 in patients with MPN (198).

## 4 Strategies to inhibit glutamine metabolism in cancer

### 4.1 Glutamine metabolism and anticancer immunity

Similar to cancer cells, activated T cells enhance glutamine uptake and metabolism to support mitochondrial anaplerosis, nucleotide synthesis, amino acid production, and redox homeostasis (199–201). Competitive depletion of glutamine by cancer cells in the TME triggers the starvation of activated T lymphocytes and suppresses their proliferation and cytokine production (202). Alternatively, glutamine deprivation impairs the T-lymphocyte-mediated anticancer immune response by promoting Treg cell activation and proliferation (203). Deleting GLS in cancer cells increases interstitial glutamine concentrations to near physiological plasma levels and enhances the overall activation and effector capacity of T lymphocytes (199, 204), suggesting that reducing glutamine confers immunosuppressive effects in the TME. Therefore, the specific inhibition of glutamine metabolism in tumor cells not only inhibits tumor growth but also improves the T cell-mediated antitumor immune response by reversing the “glutamine steal” scenario of tumors (204, 205). The glutamine-mimetic antimetabolite JHU083 disrupts metabolism in various types of tumors and reverses hypoxic, acidic, and nutrient-deprived conditions in the TME. Furthermore, it restores antitumor immunity by inducing T cell activation, extending their lifespan, and promoting memory T cell differentiation (206). These findings have confirmed glutamine metabolism as a metabolic checkpoint in cancer immunotherapy. Transient GLS inhibition can also improve the function of CAR-T cells in a mouse model administered with cellular immunotherapy (207).

### 4.2 Glutamine metabolism inhibitor

The main inhibitors of glutamine metabolism are glutamine uptake, GLS, GDH, transaminase, and GS inhibitors; glutamine-mimetic antimetabolites; and systemic glutamine-depleting drugs (**Table 2**). Most metabolic inhibitors targeting cancer remain in preclinical phases, but glutamine-depleting L-asparaginases have already been approved as a standard component of a therapeutic regimen for acute lymphoblastic leukemia (223). Additionally, the GLS inhibitor CB-839 has been used to develop monotherapy and polytherapy with chemotherapy and/or immunotherapy that have entered phase I and II of clinical trials, respectively (**Table 3**).

**Table 2:** Strategies for inhibiting glutamine metabolism.

Classification	Drug
Glutamine uptake inhibitors (ASCT2 inhibitors) LAT1 inhibitors xCT inhibitors	GPNA (208), V-9302 (209), γ-FBP (210), Benzylserine and benzylcysteine (211) JPH203 (212), KMH-233 (213) Erastin (214), Sulfasalazine (215)
Glutaminase (GLS) inhibitors Glutaminase (GLS2) inhibitor	968 (216), BPTES (217), CB-839 (115) Ardisianone (218)
Glutamate dehydrogenase (GDH) inhibitors	R162 (129), ECG and EGCG (219)
Aminotransferase inhibitor	Amino oxyacetate (140)
Glutamine synthetase (GS) inhibitor	L-methionine sulfoximine (152)
Glutamine-mimetic antimetabolites	L-DON (220), Azaserine (220), Acivicin (221), JHU083 (222)
Systemic glutamine depleting drugs	L-asparaginases (223), L-glutaminases (224), Phenylbutyrate (225)

**Table 3:** Clinical trials of glutaminase inhibitor CB-839 (Telaglenastat) against various types of cancers.

Trial identifier (ClinicalTrials.gov)	Phase and Recruitment Status	Intervention/ treatment	Disease	Outcome	Ref
NCT02071927	Phase 1, completed	CB-839 or CB-839 + Azacytidine	Acute myeloid leukemia and acute lymphocytic leukemia	CB-839 was well tolerated and robustly inhibited GLS in blood platelets and in tumors.	(226)
NCT02071888	Phase 1, completed	CB-839 or CB-839 + Dexamethasone or CB-839 + Pomalidomide + Dexamethasone	Non-Hodgkin’s lymphoma and multiple myeloma, etc	CB-839 was well tolerated and robustly inhibited GLS in blood platelets and in tumors.	(227)
NCT02071862	Phase 1, completed	CB-839 or CB-839 + Paclitaxel/Everolimus/Erlotinib/Docetaxel/Cabozantinib	Solid tumors, triple-negative breast cancer, non-small cell lung cancer renal cell carcinoma mesothelioma, etc	CB-839 showed an accep table safety profile, significant glutaminase inhibition, and preliminary signs of clinical activity in multiple tumor types.	(228–231)
NCT02771626	Phase 1/2, completed	CB-839 + Nivolumab	Melanoma, clear cell renal cell carcinoma and non-small cell lung cancer	The combination of CB-839 + nivolumab was well tolerated and disease control in MEL, ccRCC and NSCLC was encouraging.	(232)
NCT03057600	Phase 2, completed	CB-839 + Paclitaxel	Triple-negative breast cancer	Pac + CB-839 had clinical activity and was well tolerated.	(233)
NCT03163667	Phase 2, completed	CB-839 + Everolimus Placebo + Everolimus	Clear cell renal cell carcinoma	ENTRATA met its primary endpoint, supporting GLS inhibition with CB-839 as a new therapeutic approach in RCC.	(234)
NCT03263429	Phase 1/2, recruiting	CB-839 + Panitumumab + Irinotecan Hydrochloride (phase I only)	Metastatic or refractory RAS wild type colorectal cancer	Phase 1: Triplet combination was tolerable at full doses of each drug, and preliminary antitumor activity was observed in a majority of patients. Phase 2 is in progress.	(235)
NCT02861300	Phase 1/2 active, not recruiting	CB-839 + Capecitabine	Solid tumor, colorectal cancer, colon cancer, rectal cancer	Patients with PIK3CA mutant CRC experienced prolonged progression-free survival. Phase 2 is pending.	(236)
NCT03047993	Phase 1/2 active, not recruiting	CB-839 + Azacitidine	(High risk) myelodysplastic Syndrome, acute myeloid leukemia, chronic myelomonocytic leukemia	Combination treatment is a safe and effective regimen for patients with advanced MDS. Response in previously-treated and genomically high-risk patients was encouraging. The trial continues.	(237, 238)
NCT03428217	Phase 2, completed	CB-839 + Cabozantinib Placebo + Cabozantinib	Advanced or metastatic renal cell carcinoma	Combination treatment did not meet the primary end point of improved progression-free survival.	(239, 240)
NCT03965845	Phase 1/2, completed	CB-839 + Palbociclib	Solid tumors, non-small cell lung cancer, colorectal cancer, KRAS mutation	N/A	N/A
NCT03528642	Phase 1, active, not recruiting	CB-839 HCl + Temozolomide + Radiation	Astrocytoma, IDH-mutant, Grade 2 and 3	In progress	N/A
NCT03798678	Phase 1, active, not recruiting	CB-839 HCl + Carfilzomib + Dexamethasone	Recurrent or refractory plasma cell myeloma	Triplet combination was well tolerated. Ongoing correlative studies could provide mechanistic insight into which patients could benefit the most from Telaglenastat in combination with proteasome inhibitors.	(241)
NCT03831932	Phase 1/2, recruiting	CB-839 HCl + Osimertinib	Advanced or metastatic lung non-small cell carcinoma and stage IV lung cancer AJCC v8	In progress	N/A
NCT03872427	Phase 2, active, not recruiting	CB-839	Advanced malignant solid neoplasm, metastatic malignant solid neoplasm, and NF1 mutation positive malignant peripheral nerve sheath tumor, etc	In progress	N/A
NCT04250545	Phase 1, recruiting	CB-839 HCl + Sapanisertib	Metastatic or recurrent lung non-small cell carcinoma, Stage IV/IVA/IVB Lung Cancer AJCC v8	The dose finding portion of phase 1 showed the combination treatment is safe and tolerable at the recommended expansion dose. The trial continues.	(242)
NCT04265534	Phase 2, terminated	CB-839 + Pembrolizumab + Carboplatin/pemetrexed	KEAP1/NRF2/NFE2L2 mutated (non-squamous) non-small cell lung cancer	Lack of clinical benefit	N/A
NCT03944902	Phase 1, terminated	CB-839 + Niraparib	(Resistant BRCA -wild-type) ovarian cancer	The actual enrollment was only one and the participant is now off study.	N/A
NCT03875313	Phase 1/2, terminated	CB-839 + Talazoparib	Solid tumor, clear cell renal cell carcinoma, colorectal cancer, etc	The study was terminated due to slow enrollment.	N/A
NCT04824937	Phase 2, not yet recruiting	CB-839 + Talazoparib	Metastatic prostate cancer	N/A	N/A
NCT05521997	Phase 2, not yet recruiting	CB-839 + Radiation + Cisplatin	Advanced cervical carcinoma	N/A	N/A

ccRCC, clear cell renal cell carcinoma; CRC, colorectal cancer; MEL, melanoma; MDS, myelodysplastic syndrome; NSCLC, non-small cell lung cancer; RCC, renal cell carcinoma.

L-asparaginases also exhibit glutaminase activity required for durable therapeutic effects against acute lymphoblastic leukemia (243). However, glutamine depletion could cause some adverse effects such as acute pancreatitis, thrombotic complication, and immunosuppression (244). The clinical hypersensitivity led to the development of L-asparaginases with different bacterial origins (245). Human-derived L-asparaginase may be a solution in the future considering the problems of immunosuppression and hypersensitivity. Reversible and asymptomatic elevations in transaminases were the primary adverse effects when CB-839 monotherapy was used to treat hematologic malignancies and solid tumors in clinical trials (226–228). On balance, CB-839 was well tolerated and produced robustly inhibition of GLS in blood platelets and in tumors. Targeted nano-delivery systems have been developed to further improve antitumor efficacy and reduce systemic effect. CB-839 loaded nanoparticles could preferentially accumulate in tumor tissue through enhanced penetration and retention (EPR) effect, known as passive targeting (246). Furthermore, ligand/antibody-modified nanoparticles could recognize overexpressed tumor cell receptor/antigen, which actively targets tumor glutamine metabolism and achieves tumor-specific accumulation of CB-839 (247).

## 5 Concluding remarks and future perspectives

Many dogmas have been overturned and refined since the discovery of oncogenic metabolic alterations and the rediscovery of the roles played by glutamine and glucose in cancer cell proliferation. Here, we provide an overview of the uptake, transport and metabolism of glutamine, as well as the regulation of glutamine addiction by oncogenes in cancer. The oncogene-driven types of cancer summarized herein are highly dependent on glutamine. Hence, targeting glutamine metabolism could facilitate the pharmacological improvement of cancer therapeutics. In contrast, some oncogenic drivers could allow cancer cells to bypass the need for glutamine by upregulating other metabolic pathways for their cellular growth and proliferation. However, targeted inhibition of some oncogenic drivers can restore cellular reliance on glutamine. Therefore, inhibiting glutamine metabolism and these oncogenic drivers could collectively induce synthetic lethality in cancer cells.

The potent ability of glutamine metabolic inhibitors to enhance the anticancer immune response may be a feasible mechanism through which their therapeutic effectiveness can be improved. Thus, the most favorable glutamine-blocking strategy should be considered. The latest studies show that immune checkpoint blockades exhibit synergistic effects with glutamine uptake inhibitors (248, 249), GLS inhibitor (250), and glutamine-mimetic antimetabolites (206, 251). Additionally, nanowire sensors can be used to monitor changes in the level of cancer-associated proteins and mRNAs (252). Therefore, real-time dynamic monitoring of intratumoral metabolic processes may enable dynamic optimization or adjustment of therapeutic strategies, which is an important step towards developing precision medicine against cancers.

To date, the outcomes of clinical trials using glutamine metabolism inhibitors to treat cancer remain unsatisfactory because of the metabolic plasticity exhibited by cancer cells. Nevertheless, a scientific foundation has been lain for the further assessment of potential targeted molecules and the rational design of polytherapies to maximize clinical efficacy.

## Author contributions

Idea and design: RN. Evidence collection, analysis, and arrangement: RN, ZL and LiL. Manuscript drafting: RN and ZL. Critical revision of the manuscript: LinL and YL. Obtained funding: YL. Technical support: DP and YM. All authors contributed to the article and approved the submitted version.
